# Characterization of Fructose-1,6-Bisphosphate Aldolase during Anoxia in the Tolerant Turtle, *Trachemys scripta elegans*: An Assessment of Enzyme Activity, Expression and Structure

**DOI:** 10.1371/journal.pone.0068830

**Published:** 2013-07-18

**Authors:** Neal J. Dawson, Kyle K. Biggar, Kenneth B. Storey

**Affiliations:** Institute of Biochemistry & Department of Biology, Carleton University, Ottawa, Ontario, Canada; Mayo Clinic, United States of America

## Abstract

One of the most adaptive facultative anaerobes among vertebrates is the freshwater turtle, *Trachemys scripta elegans*. Upon a decrease in oxygen supply and oxidative phosphorylation, these turtles are able to reduce their metabolic rate and recruit anaerobic glycolysis to meet newly established ATP demands. Within the glycolytic pathway, aldolase enzymes cleave fructose-1,6-bisphosphate to triose phosphates facilitating an increase in anaerobic production of ATP. Importantly, this enzyme exists primarily as tissue-specific homotetramers of aldolase A, B or C located in skeletal muscle, liver and brain tissue, respectively. The present study characterizes aldolase activity and structure in the liver tissue of a turtle whose survival greatly depends on increased glycolytic output during anoxia. Immunoblot and mass spectrometry analysis verified the presence of both aldolase A and B in turtle liver tissue, and results from co-immunoprecipitation experiments suggested that in the turtle aldolase proteins may exist as an uncommon heterotetramer. Expression levels of aldolase A protein increased significantly in liver tissue to 1.59±0.11-fold after 20 h anoxia, when compared to normoxic control values (*P*<0.05). A similar increase was seen for aldolase B expression. The overall kinetic properties of aldolase, when using fructose-1,6-bisphosphate as substrate, were similar to that of a previously studied aldolase A and aldolase B heterotetramer, with a K_m_ of 240 and 180 nM (for normoxic and anoxic turtle liver, respectively). Ligand docking of fructose-1,6-bisphosphate to the active site of aldolase A and B demonstrated minor differences in both protein:ligand interactions compared to rabbit models. It is likely that the turtle is unique in its ability to regulate a heterotetramer of aldolase A and B, with a higher overall enzymatic activity, to achieve greater rates of glycolytic output and support anoxia survival.

## Introduction

Living animals are constantly faced with various environmental stresses that challenge normal life, such as oxygen and water limitation, very low or high temperatures and food restriction [1]. Of these, oxygen variation in the environment is one that many animals commonly experience. Change in oxygen supply can arise by either variations in environmental oxygen availability (e.g. ice-locked ponds and lakes with hypoxic or anoxic water) that deny organisms access to oxygen for extended periods of the time, or by animal behaviors that interrupt the supply of oxygen (e.g. breath-hold diving by lung-breathing animals, aerial exposure of gill-breathers). A decrease in oxygen supply leads to an inhibition of oxidative phosphorylation and a subsequent interruption of mitochondrial ATP production. Decreases in ATP production can quickly lead to a disruption of many ATP-utilizing processes in the cell and, if prolonged, can induce cellular death [2]. One of most adaptive facultative anaerobes among vertebrates is the freshwater turtle, *Trachemys scripta elegans*. These turtles are able to survive without oxygen for up to 18 weeks when submerged in cold water (3°C) [3], [4]. In contrast, mammalian tissues are notoriously sensitive to even brief episodes of anoxia, whereas turtle tissues are able to survive by: (1) rapidly decreasing their metabolic rates to ∼10% of normoxic resting rates, (2) increasing metabolic fuel by utilizing stores of glycogen loaded into all organs and (3) buffering and storing the lactic acid produced by anaerobic glycolysis in their bony shell [4], [5].

Upon encountering hypoxia, the first response of the turtle is to enhance oxygen delivery and extraction systems. These mechanisms are well established in the literature and include: the physiological responses of increasing lung ventilation, alterations to hemoglobin oxygen affinity, as well as increasing cardiac output to improve oxygen delivery to organs [6], [7]. If oxygen concentrations continue to fall, the systemic alterations to oxygen extraction quickly become inadequate to supply enough oxygen to deprived tissues. Oxygen-independent metabolic pathways, such as anaerobic glycolysis, are then fully recruited and are followed by cellular alterations to reduce oxygen demand and ATP [6]–[8].

Previous studies have explored the importance of regulating glycolytic enzymes during anoxia exposure in *T. s. elegans* [9]–[11]. These studies have documented significant changes in both enzyme kinetics and activity upon prolonged exposure to anoxia. Regulated liver enzymes involved in glycolysis include: phosphofructokinase (PFK), demonstrating a 3-fold decrease in the I_50_ value for citrate and a 1.5-fold increase in the K_m_ value for ATP during anoxia [9]; pyruvate kinase (PK), which demonstrated an anoxia responsive 1.5-fold increase in the I_50_ for alanine and a 1.2-fold increase in maximal activity [9]; lactate dehydrogenase (LDH), displaying an increase in the K_m_ value for pyruvate at various temperature and pH conditions, a decrease in the maximal activity (1.6-fold) in the lactate producing direction and a decrease in the I_50_ for pyruvate at various temperature and pH conditions during exposure to anoxia [10]. Furthermore, a comprehensive study of 20 enzymes involved in intermediary metabolism showed multiple changes in activities due to prolonged exposure to anoxia [11]. These studies suggest that global control of glycolytic enzymes during bouts of anoxia seem to be paramount in the adaptation of *T. s. elegans* to anoxic stress. More specifically, glycolytic control appears to be tightly regulated during anoxia. However, it would seem that major glycolytic enzymes are independently regulated and an in-depth exploration of each glycolytic enzyme should provide a more clear and robust understanding of the complex mechanisms by which the turtle can survive prolonged periods of oxygen deprivation.

Within the glycolytic pathway, fructose-1,6-bisphosphate (FBP) aldolase cleaves FBP to glyceraldehyde-3-phosphate (G3P) and dihydroxyacetone phosphate (DHAP), facilitating increase anaerobic production of ATP and lactate. Aldolase enzymes are known to be divided into two major classes: 1) class I aldolases form a Schiff-base complex with FBP at a lysine residue in the active site, have a molecular weight of approximately 160 kDa, are homotetramers, and are found in animals, plants and protozoa, and 2) class II aldolases employ the use of divalent metal ions, have molecular weights of approximately 80 kDa, are dimeric, and are primarily found in bacteria and fungi [12]. In animals, there have been three types of class I aldolases discovered; aldolase A found in the muscle, aldolase B in the liver, and aldolase C in brain [13]. There are also examples of heterotetramers comprising more than one type of subunit from A, B, and/or C aldolases [14]. Interestingly, turtle liver has been previously reported to contain both aldolase A (ALDOA) and aldolase B (ALDOB) as a hybrid heterotetramer comprising one, two, or three of either A or B parental subunit(s) [15]. It has also been reported that individual ALDOA subunits have approximately 50× greater enzymatic activity than equivalent ALDOB subunits [16]. Therefore, the hybrid forms of liver aldolase can exhibit overall higher aldolase activity than that of homotetramer ALDOB activity. Each additional ALDOA monomer present in the tetramer stoichiometrically contributes to greater overall enzymatic activity [16]. It is interesting to speculate that perhaps the presence of a hybrid heterotetramer of aldolase could help adapt the turtle to the higher glycolytic demands of anoxia survival.

Additionally, the enzymatic activity of aldolase can be significantly altered by changes to protein structure. The importance of structure and stability to the function of aldolase has been previously demonstrated with the use of both artificially derived and natural mutants of aldolase protein [17]. Alterations in aldolase structure and enzymatic activity have been widely studied as a result of their implications for fructose intolerance, a condition that can be attributed to small changes in protein structure [18]. Such alterations in structure have been shown to have significant impact on both the kinetic parameters [19] and the stability of the catalytically active dimer [17]. Due to the importance of aldolase for fructose metabolism, the present study aims to explore structural changes in aldolase protein that may confer advantageous changes in enzyme function in the anoxic turtle.

Much is known about the molecular responses of many organisms to various environmental stressors and disease states, particularly those pertaining to maintaining human health. These studies have historically developed a general, but refined, view of the important molecular pathways contributing to any particular stressor. However, this type of research is currently undergoing a rapid period of growth as new technologies are developed, allowing deeper analysis of any individual protein. This study uses several classical (column chromatography, immunoblotting and immunoprecipitation) and modern (mass spectrometry and protein modeling) molecular techniques to characterize aldolase activity and structure from the liver tissue of an anoxia tolerant turtle, *T. s. elegans.* The study also explores possible mechanisms and structural adaptations of aldolase and how its regulation may aid survival during periods of anoxia.

## Materials and Methods

### Ethics Statement

All animals were cared for in accordance with the guidelines of the Canadian Council on Animal Care and all experimental procedures had the prior approval of the Carleton University Animal Care Committee.

### Animal Treatment and Chemicals

Adult female red-eared sliders (*T. s. elegans*), 700–1500 g, were acquired from local suppliers and held at 5±1°C in large plastic tubs (2 turtles per tub) filled with dechlorinated tap water for several days before use. Normoxic turtles were sampled from this condition. For anoxia exposure, turtles were transferred to large buckets at 5±1°C that had been previously bubbled with N_2_ gas for 1 h; 2–3 turtles were added per bucket in 30 min intervals. Bubbling was continued for 1 h after the last turtle was added and was re-initiated again during sampling of the animals. A wire mesh was fitted into the tank about 5 cm below the water surface so that turtles remained submerged throughout a 20 h exposure to experimental anoxia. All animals were immediately sacrificed after 20 h anoxic exposure by decapitation and tissues were rapidly dissected out, frozen in liquid nitrogen and stored at −80°C until use.

Chemicals, biochemicals, chromatography media and coupling enzymes were from Sigma Chemical Co. (St. Louis, Missouri, United States). The primary ALDOA and ALDOB antibodies were purchased from GeneTex (Cat# GTX37468 and GTX101363, respectively). Secondary anti-rabbit IgG HRP-conjugated antibody was purchased from Cell Signaling (Cat# 7074). Primary α-tubulin antibody was purchased from Developmental Studies Hybridoma Bank at the University of Iowa (Cat# 12G10). All antibodies were prepared according to manufacturer’s specifications.

### Immunoblotting

Samples of frozen liver tissue were crushed under liquid nitrogen and then homogenized 1∶2.5 w:v in homogenizing buffer A (20 mM Hepes, pH 7.5, 200 mM NaCl, 0.1 mM EDTA, 10 mM NaF, 1 mM Na_3_VO_4_, 10 mM β-glycerophosphate) with a few crystals of phenylmethylsulfonyl fluoride (PMSF) and 1 µL/mL Sigma protease inhibitor cocktail (104 mM AEBSF, 80 µM Aprotinin, 4 mM Bestatin, 1.4 mM E-64, 2 mM Leupeptin, 1.5 mM Pepstatin A). Samples were homogenized on ice with a Polytron PT1000 homogenizer and were then centrifuged at 4°C for 15 min at 10,000×*g*. Soluble protein concentrations were assessed using the BioRad protein assay using bovine serum albumin (BSA) as the standard. All samples were adjusted to a constant concentration by adding a calculated small volume of buffer A. The samples were then mixed 1∶1 v:v with 2× SDS loading buffer (100 mM Tris-base, 4% w/v SDS, 20% v/v glycerol, 0.2% w/v bromophenol blue, 10% v/v 2-mercaptoethanol). Final sample concentrations were 5 µg/µl. Proteins were denatured by placing the tubes in boiling water for 5 min. Samples were stored at −80°C until use.

Aliquots containing 20 µg of total soluble protein from liver tissue were loaded onto 10% polyacrylamide gels together with prestained molecular weight standards (FroggaBio; Cat# PM005-0500) and separated using a discontinuous buffer system. Electrophoresis was carried out at 180 V for 1 h using the BioRad Mini-Protean 3 system with 1× Tris-glycine running buffer. Proteins on the gel were then electroblotted onto polyvinylidene difluoride (PVDF) membrane (Millipore, Bedford, MA) using a BioRad mini Trans-Blot cell. The transfer was carried out at 160 mA constant amperage for 1.5 h. Following the transfer, membranes were washed in TBST (10 mM Tris, pH 7.5, 150 mM NaCl, 0.05% v/v Tween-20) for 3×5 min. The membranes were blocked using 2.5% skimmed milk in TBST for 1 h. After blocking, the membranes were probed with primary antibody diluted 1∶1000 v:v for 24 h at 4°C. The membranes were washed 3×5 min with TBST at room temperature (RT) and probed with secondary antibody (1∶4000 v:v dilution) for 1 h. Membranes were washed again 3×5 min in TBST at RT and were then developed using SuperSignal West Pico chemiluminescence substrate (Pierce; Cat#34079) and visualized using a Chemigenius (Syngene, Frederick, MD).

The GeneTools program was used to quantify the protein bands. Statistical testing used the Student’s t-test (*P*<0.05). Both ALDOA and ALDOB cross-reacted with a band corresponding to a molecular weight of ∼37 kDa. Immunoblotting with α-tubulin antibodies showed constant α-tubulin expression in both control and experimental conditions and when compared with the combined density of a group of Coomassie stained protein bands. Immunoblots of the proteins of interest were individually adjusted for loading irregularities by normalizing the band intensity of the immunoreactive material in each lane against the combined density of a group of Coomassie stained protein bands in its respective sample lane; these bands showed a similar expression pattern to α-tubulin, and the same stained bands were used for such comparison.

### Co-immunoprecipitation

Samples of frozen liver tissue were crushed under liquid nitrogen and then homogenized 1∶4 w:v in homogenizing buffer B (10 mM Tris HCl, pH 7.4, 10 mM NaCl, 2 mM EDTA, 0.1% Triton X, 0.5 mM dithiothreitol (DTT), 10 mM β-glycerophosphate) with a few crystals of PMSF and 1 µL/mL of Sigma protease inhibitor cocktail. Samples were homogenized on ice with 20 strokes of a Dounce homogenizer and incubated for 20 min. Samples were then centrifuged at 4°C for 15 min at 10,000×*g*. Soluble protein concentrations were assessed using the BioRad protein assay using BSA as the standard. All samples were adjusted to a constant concentration by adding a calculated small volume of buffer B.

For co-immunoprecipitation (co-IP), cleared samples containing 200 µg of total protein (1 µg/µl) were rotated at 4°C overnight with 5 µl of ALDOB capture antibody (0.59 mg/mL). After incubation with capture antibody, samples were rotated with 20 µL of prepared Protein A-agarose at 4°C for 4 h (Santa Cruz; Cat# sc-2001). The immunoprecipitation complex was then washed three times with wash buffer (10 mM Tris HCl, pH 7.4, 10 mM NaCl, 0.05% Triton X), and protein complexes were eluted in 2× SDS loading buffer. Aliquots containing 20 µl of sample from ALDOB co-IP preparations were loaded onto 12% polyacrylamide gels together with prestained molecular weight standards (FroggaBio; Cat# PM005-0500). Co-IP samples were subjected to immunoblotting as previous described for the presence of either ALDOA or ALDOB.

### Protein Preparation and Purification of Aldolase

For protein purification, samples of frozen liver tissue were homogenized 1∶5 w:v in ice-cold homogenizing buffer C (20 mM potassium phosphate [KPi] buffer, pH 7.2 containing 15 mM β-glycerophosphate, 1 mM EGTA, 1 mM EDTA, 15 mM β-mercaptoethanol, 5% glycerol and 1 mM PMSF). Homogenates were then centrifuged at 13,500×*g* at 4°C and the supernatant was decanted. A 3 mL aliquot of homogenate was then applied to a DEAE^+^ column (1.5 cm×10 cm), previously equilibrated in buffer C (30 mL of the buffer was passed through the column). Aldolase did not bind to the DEAE^+^ column, however, >50% of the total protein was removed during this chromatography step. A total of four fractions, each containing 4 mL of sample, were collected and 5 µL of each fraction were assayed for aldolase activity (see below for methodology). The fractions of peak aldolase activity were pooled and applied to a Cibacron Blue 3GA column (1.5 cm×5 cm), equilibrated in buffer C. The column was then washed with 30 mL of buffer C to remove unbound protein. Bound proteins were eluted with 60 mL of 5 mM FBP in buffer C. A total of 23 fractions, each containing 1275 µL of sample, were collected and 10 µL of each fraction were assayed for aldolase activity. Fractions containing the highest aldolase activity from the Cibacron Blue 3GA column were pooled, diluted 20× in buffer C, and subjected to a second Cibacron Blue 3GA column (1.5 cm×5 cm), equilibrated in buffer C. The column was washed with 30 mL of buffer C and then bound proteins were eluted with a linear KCl gradient (0–2 M over a volume of 40 mL). A total of 11 fractions, each containing 850 µL of sample, were collected and 10 µL from each fraction were assayed for aldolase activity. Fractions containing the highest activity were combined and used for further study. The purity of aldolase was determined by combining samples with equal volume of 2× SDS loading buffer, boiling for 5 min, then running 20 µl of sample purified from each successive purification step on SDS-PAGE (as previously described). Protein banding was identified by post-run gel staining with Coomassie blue.

### Aldolase Enzyme Activity Assay

One unit of aldolase activity is defined as the amount of enzyme that cleaved 1 µmol of FBP/F1P per minute, as outlined in Nakamura et al. [20]. Optimal assay conditions for purified aldolase were: 100 mM KPi (at pH 7.2), 1 mM FBP, 10 mM EDTA, 2 mg/ml α-glycerophosphate dehydrogenase, 2 mg/ml triose phosphate isomerase, 100 µg/ml BSA, 0.15 mM NADH and 10 µl of purified enzyme extract for a total volume of 200 µL. Enzyme activity was assayed using a Thermo Labsystems Multiskan spectrophotometer at 340 nm. One unit of aldolase activity is defined as the amount of enzyme that produced 1 µmol of NADH per minute at 25°C. Data were analyzed using the Kinetics v.3.5.1 program. Statistical testing used the Student’s t-test (*P*<0.05). Protein concentrations were assessed using the BioRad protein assay using BSA as the standard.

### Protein Digestion and Mass Spectrometry

Purified proteins were subjected to a 10% SDS-polyacrylamide gel and resolved using a discontinuous buffer system similar to the previously mentioned protocol. The isolated proteins were then cut from the SDS-polyacrylamide gel and washed three times with 1 mL of 50 mM ammonium bicarbonate (pH 7.8). Samples were briefly centrifuged and wash ammonium bicarbonate solution discarded after each wash step. These fragments were dehydrated with 50 µL of 50% (v/v) acetonitrile and 25 mM ammonium bicarbonate solution (pH 7.8) for 15 min at RT. Samples were briefly centrifuged and the liquid discarded. Samples were then dried completely using a speed vacuum for 20–25 min. In separate stages, the gel fragments were reduced with 30 µL of 10 mM DTT and 50 mM ammonium bicarbonate (pH 7.8) and incubated at 56°C for 15 min. Samples were briefly centrifuged, liquid discarded and then alkylated with 100 mM iodoacetamide and 50 mM ammonium bicarbonate (pH 7.8) for 15 min in the dark at RT. The gel pieces were washed in 50 mM ammonium bicarbonate (pH 7.8) and then dehydrated again as previously described. The protein samples were then digested with 28 µL of a 50 mM ammonium bicarbonate solution (pH 7.8) supplemented with 0.36 µg of sequencing grade porcine trypsin (Promega, WI). After full absorption, 10 µL of 25 mM ammonium bicarbonate solution was added and samples were incubated at 37°C for 3 h. Following this incubation, samples were briefly centrifuged and the solution was transferred to a separate clean sample container. To the gel pieces, 50 µL of 25 mM ammonium bicarbonate (pH 7.8) was added and samples were incubated for 20 min. Following incubation, samples were centrifuged and liquid was combined with the solution collected from the previous step. Finally, peptides were extracted by incubating each gel sample with 50 µL of 5% formic acid and 50% acetonitrile for 20 min. Samples were centrifuged and liquid combined with the respective pooled sample from the previous steps. Extracted peptides were concentrated using a speed vacuum until dryness was achieved (20–25 min). Peptides were then resuspended in 40 µL of 0.1% formic acid and used immediately for subsequent mass spectrometry.

Peptides were loaded onto a 5 µm×50 mm column packed in-house with 5 µm Magic C18AQ beads (300 Å pore size) (Michrom Bioresources, CA) at a rate of 20 µL/min using a Dinoex 3000 ultimate HPLC system (Thermo Fisher Scientific, IL). Peptides were washed with an aqueous solution containing 5% acetonitrile and 0.1% formic acid. Next, peptides were eluted from the column at a flow rate of 250 nL/min and analyzed using an AB SCIEX 4000 QTRAP Hybrid Triple Quadrupole ion trap mass spectrometer (ABI/MDS Sciex, ON). The HPLC pumped 0.1% formic acid in water with the following percentage gradient of acetonitrile: 0 min: 5%, 3 min: 15%, 10 min: 40%, 15 min: 50%, and 20 min: 80% and a final wash step at 100%.

### Bioinformatic Analysis

The recorded MS/MS spectra were searched against the ‘boney vertebrates’ taxonomic grouping in the NCBInr database using Mascot (v.2.3) (Matrix Science Ltd., MA). The Mascot peptide and MS/MS tolerances for the experiments were set to ±1.3 Da and ±0.6 Da for the peptide and fragment ion spectra, respectively; two missed cleavage sites were also considered. As the tryptic digestion involved an alkylation step, carbamindomethyl was considered a fixed modification. Variable modifications selected included: acetylation (K), methylation (DE), oxidation (M) and phosphorylation (Y and ST). All matches from the database were manually verified to contain at least three successive y- or b- ions. The predicted tertiary and quaternary structures of matched proteins were created using the SWISS-MODEL server (http://swissmodel.expasy.org/) [21]. Crystal structures used for protein modeling were from rabbit (*Oryctolagus cuniculus*) aldolase A (muscle-type) and B (liver-type) (PDB entries 1zaiA and 1fdjA, respectively) [22], [23]. As all predicted structures are heterologous (not matched to host turtle species), ProQ-Protein quality predictor scores were used to validate the model [24]. Next, all protein models were protonated and optimized by energy minimization using MMFF94× force field model in Molecular Operating Environment (MOE) software (v.2011.10) (Chemical Computing Group, QC).

### Docking

The ligand binding coordinates in FBP aldolases were identified from crystal structures of rabbit aldolase A (muscle-type) and B (liver-type) in complex with FBP ligand (PDB entries 1zaiA and 1fdjA, respectively) [22], [23]. Next, the structure of FBP was docked to the active site of each studied aldolase using MOE Dock, employing Triangle Matcher as the placement method and the function London dG as the first scoring function. The refinement was set to force field and the docked poses were energy minimized in the receptor pocket. The final refined poses were ranked via MM/GBVI binding free-energy estimation (E_refine score). Thirty poses were constructed for FBP in all protein models, the best scoring model-ligand complexes were selected; the ligand interactions within these complexes were visualized using MOE Ligand Interactions simulation.

## Results

### Aldolase Purification and Expression

**Figure 1:**
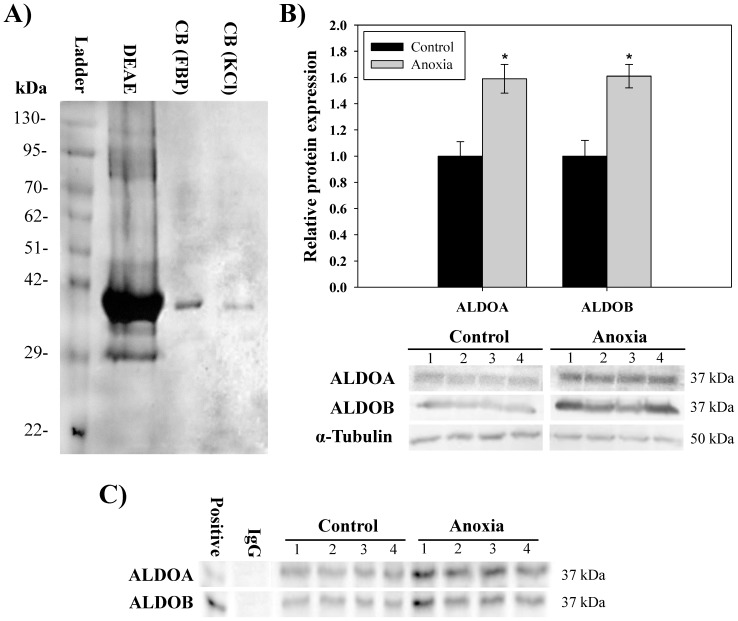
Aldolase purification and expression from the liver of control and anoxic turtles. A) SDS-PAGE gel with Coomassie blue staining of samples taken at each stage of aldolase purification from normoxic turtles *T. s. elegans*. B) Effect of 20 h of anoxic submergence on relative ALDOA and ALDOB total protein expression demonstrated with immunoblotting on whole cell lysates from liver tissue. Histogram showing mean normalized protein expression. Data in histograms are means ± SEM, n = 4 independent trials with tissue from different animals. * - indicates significantly different from the corresponding control (*P*<0.05). C) Co-immunoprecipitation of ALDOA and ALDOB from turtle liver. Endogenous ALDOA was co-immunoprecipitated with endogenous ALDOB in control and 20 h anoxic conditions. Experimental IgG control was carried out using p130 primary antibody (Santa Cruz; Cat# sc-317).

The purification scheme used for turtle liver aldolase is shown in Table 1. The procedure used ion exchange chromatography on DEAE^+^ Sephadex followed by two rounds of affinity chromatography by Cibacron Blue (CB) (Figure S1). Liver aldolase was purified 363-fold from total protein extracts, with an overall 21% yield of enzymatic activity. The final specific activity of the purified enzyme was 22.8 U/mg of protein. The purification of aldolase was assessed using SDS-PAGE with the gel stained with Coomassie blue (Figure 1A). The polyacrylamide gel showed a highly purified protein band corresponding to the expected molecular weight of 37 kDa for aldolase [9]. Immunoblotting of endogenous ALDOA and ALDOB was used to assess expression in turtle liver tissue (Figure 1B). Expression level of ALDOA protein increased significantly in liver tissue to 1.59±0.11-fold after 20 h anoxia when compared to normoxic control values (*P*<0.05). A similar increase of 1.61±0.09-fold was seen for ALDOB protein expression in response to 20 h anoxia exposure when compared to normoxic control values (*P*<0.05) (Figure 1B). Co-immunoprecipitation analysis was used to assess whether heterotetramers of aldolase exist in turtle liver and whether ALDOA/ALDOB ratios change in response to anoxia. Immunoprecipitation of ALDOB successfully co-purified ALDOA in turtle liver from both control and 20 h anoxic conditions (Figure 1C). No significant change was observed in the ratio of ALDOA bound to ALDOB in response to 20 h anoxia exposure when compared to control values. Co-immunoprecipitation for the ALDOA antibody used in this study was not recommended by the manufacturer and reverse co-IP experiments for ALDOA were unsuccessful.

**Table 1:** Outline of aldolase purification from liver of *T. s. elegans*.

	Total protein (mg)	Total activity (U)	Specific activity (U/mg)	Fold purification	Yield (%)
Crude	36	2.3	0.06	–	–
DEAE+	15	2.0	0.13	2	86
Cibacron Blue (FBP)	0.3	0.98	3.4	55	44
Cibacron Blue (KCl)	0.02	0.48	23	363	21

### Aldolase Kinetic Parameters

**Figure 2:**
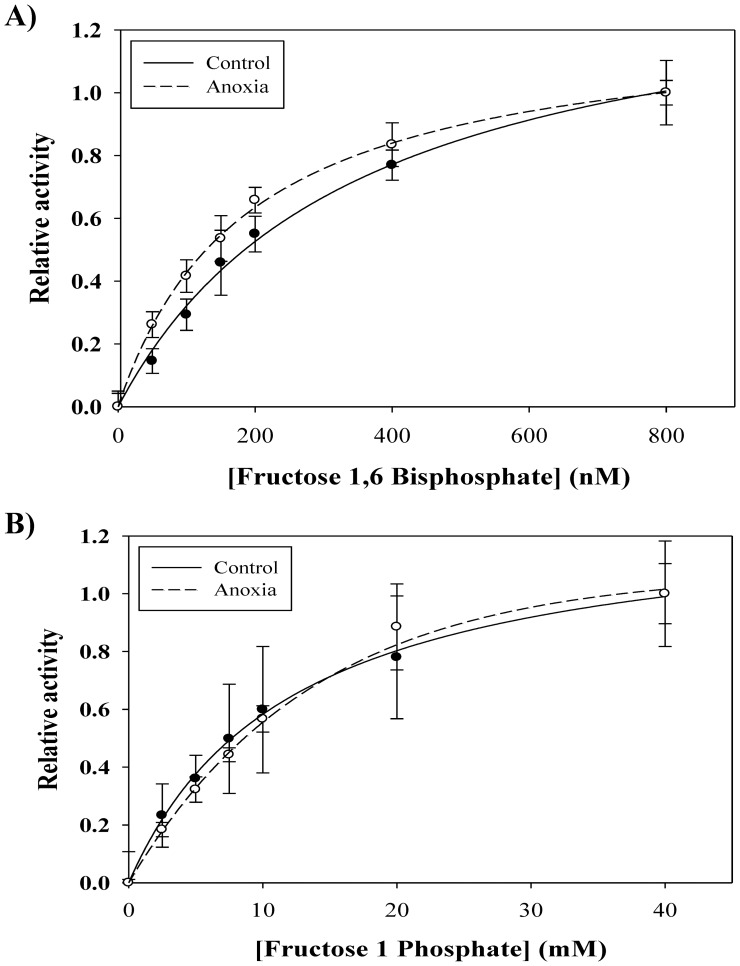
Kinetic parameters of purified aldolase from the liver of control and anoxic turtles. Representative kinetic plots showing the Michaelis-Menten constants of normoxic and anoxic aldolase with respect to A) FBP and B) F1P concentration.

Kinetic parameters of purified liver aldolase were assessed to detect any enzymatic differences between normoxic and anoxic conditions (Figure 2). Kinetic analysis determined a significant decrease in the K_m_ of aldolase for FBP during anoxia. After 20 h anoxia, the K_m_ decreased from 240±30 nM in normoxic controls to 180±20 nM, a decrease to 75±11% of normoxic values (*P*<0.05) (Figure 2A). The K_m_ value for fructose-1-phosphate (F1P) did not change significantly between control and stressed conditions (Figure 2B). The V_max_ for FBP was observed to have no significant difference between normoxic (63±4 U/g wet weight) and anoxic (56±3 U/g wet weight) conditions. Similarly, there was no observed difference of the V_max_ value associated with F1P between normoxic (17±2 U/g wet weight) and anoxic (20±2 U/g wet weight) conditions. The ratio between FBP/F1P aldolase activity was found to be 3.68 for normoxic turtles and 2.86 for anoxic turtles; these values were not significantly different (*P*<0.05). This suggests that turtle liver aldolase favors FBP as a substrate and is unchanged between normoxia and anoxia.

### Mass Spectrometric Analysis

**Figure 3:**
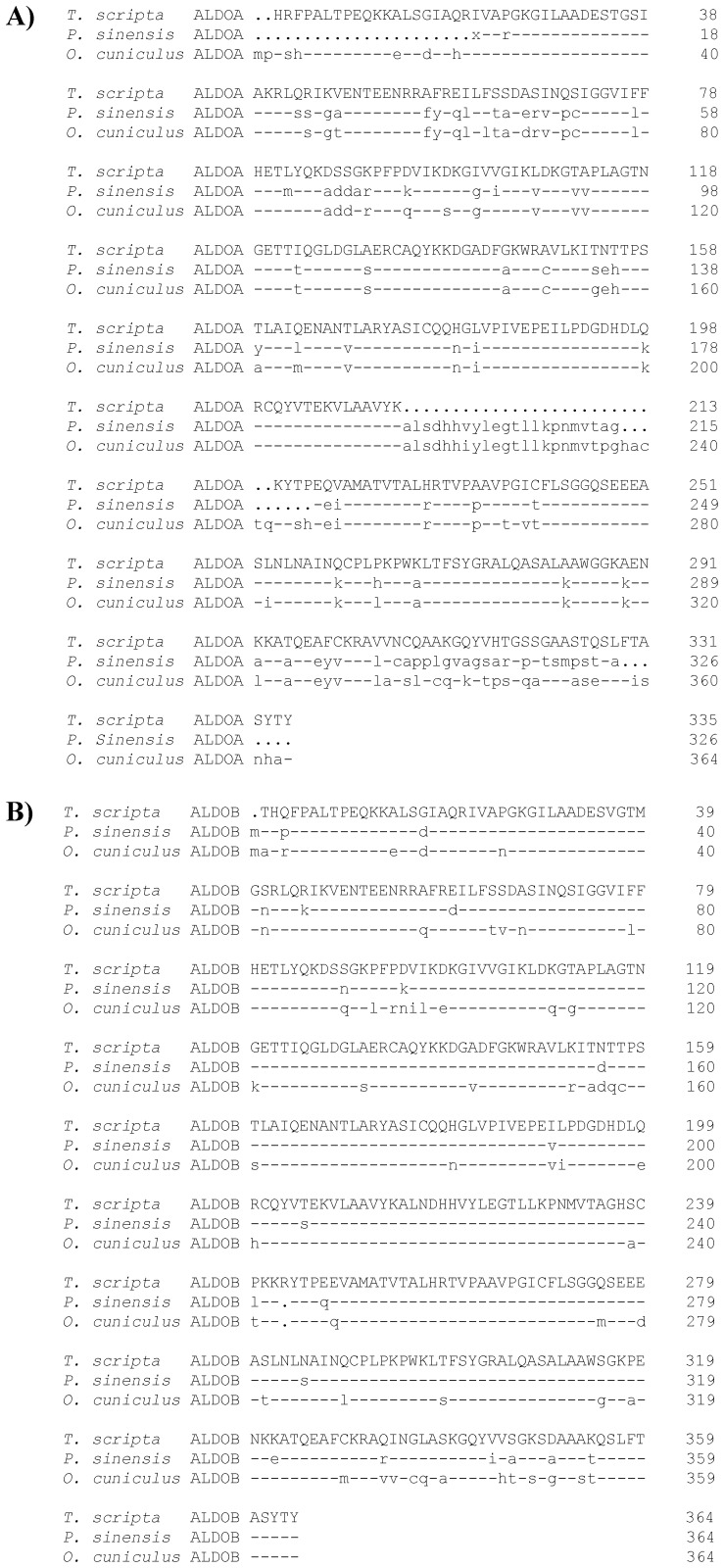
Protein alignment of ALDOA and ALDOB. Full amino acid sequences of A) ALDOA and B) ALDOB from *T. s. elegans* compared to *O. cuniculus* and *P. sinensis*. Dashes replace amino acid residues identical with *T. s. elegans*; spacer dots indicate residues that are missing in the sequence. Alignment was prepared using DNAMAN (v.4) software.

Using Mascot (v.2.3), peptides from three proteins were identified from the purified aldolase samples of turtle liver. We determined that two of these proteins were ALDOA and ALDOB, while the remaining protein was an autodigestion of Trypsin. The MASCOT identification of these proteins are presented in Table 2 and includes GenBank accession number, protein name, organism of closest match, Mascot score, number of peptide hits (and unique peptide hits), and percent protein coverage of the peptide hits. Percent protein coverage of the peptide hits were calculated by dividing the number of amino acid residues spanned from the identified peptides by the total number of amino acid residues. The full length protein sequences of turtle specific ALDOA and ALDOB were identified from *T. s. elegans* genome supercontigs (v.1.0) and aligned with the protein sequences from *O. cuniculus* (UniProtKB entries P00883 and P79226, respectively) and *Pelodiscus sinensis* (UniProtKB entries K7GIC4_PELSI and K7FNK8_PELSI, respectively) using DNAMAN (v.4) software (Figure 3).

**Table 2:** List of all proteins identified by Mascot from mass spectrometry data.

Accession	Protein	Organism	Mascot score	Peptides (#)	Coverage (%)
XP_003216643	Aldolase B	*A. carolinensis*	382	6	37
IFDJ_A	Aldolase A	*O. cuniculus*	214	5	31
P00761	Trypsin	*S. scrofa*	164	3	21
XP_003405511	Keratin (type II)	*L. africana*	127	3	5
AAA37265	GOT2	*M. musculus*	98	2	3

Identified proteins are from whole cell liver lysates of normoxic turtle, *T. s. elegans*.

### Prediction of Protein Structure and Ligand Binding

**Figure 4:**
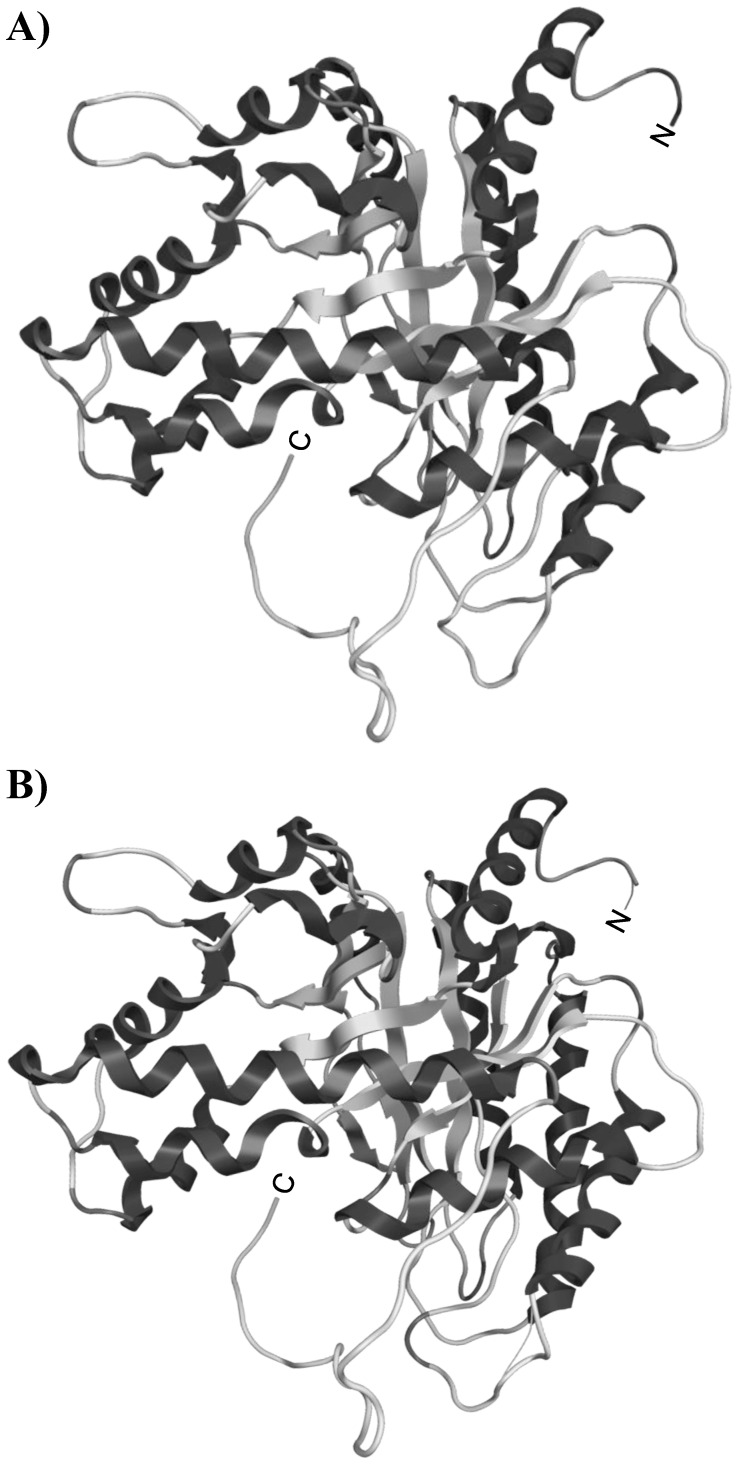
Predicted tertiary structures of ALDOA and ALDOB from the turtle, *T. s. elegans*. Structures of A) ALDOA and B) ALDOB were predicted using SWISS-MODEL with Rabbit aldolase as reference (1zaiA and 1fdjA, respectively). Structures were further optimized with MOE (v.2011.10), protonated and folding energy minimized. Both structures were validated using ProQ-protein quality predictor.

**Figure 5:**
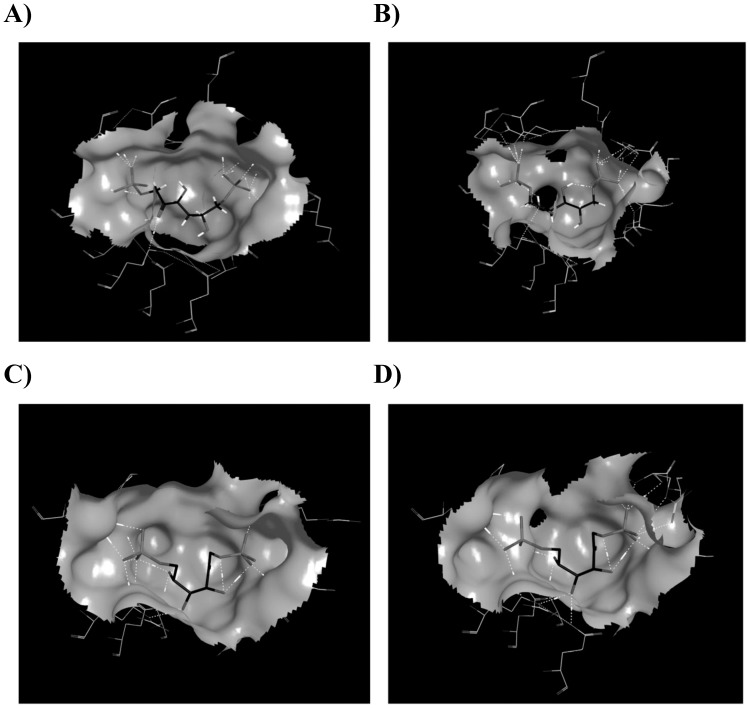
Predicted docking of FBP substrate in the active site of aldolase. Best-scoring ligand docking complexes for ALDOA in the A) turtle and B) rabbit (crystal structure 1zaiA), as well as, ALDOB in the C) turtle and D) rabbit (crystal structure 1fdjA). Docking was modeled using MOE Dock, employing Triangle Matcher as the placement method and the function London dG as the first scoring function.

Molecular models were predicted from the primary sequences of both ALDOB and ALDOA from the *T. s. elegans* genome supercontigs (v.1.0). Models were created based on the *O. cuniculus* ALDOB and ALDOA templates (PDB entries 1fdjB and 1fdjA, respectively) by the SWISS-MODEL automated protein structure homology modeling server (Figure 4). Both ALDOB (LGscore = 2.72) and ALDOA (LGscore = 4.15) models were validated using ProQ-protein quality predictor where LGscores greater than 2.5 indicate very good predictions. Finally, all protein models were protonated and optimized by energy minimization using MMFF94s forcefield model in MOE software. Predicted FBP ligand docking structures are presented in Figure 5. Docking structures for turtle aldolase (with FBP ligand) displayed similar docking energetics and degree of H-bonding between protein and ligand when compared to the respective rabbit crystal structure (Figure S2).

## Discussion

Various species of freshwater turtles have well-developed tolerances for anoxia. Survival times of up to 18 weeks at 3°C have been reported for *T. s. elegans* [7]. Metabolic adaptations supporting such facultative anaerobiosis include: 1) large reserves of fermentable substrate (glycogen), particularly in liver, along with substantial activities of glycolytic enzymes in all organs, and 2) strong metabolic rate depression. Anaerobic glycolysis, converting hexose phosphates to lactate, supports the ATP requirements of all organs of the anoxic turtle. As a result, there is strict regulatory control over glycolysis which is critical for the maintenance of homeostasis during long-term anaerobiosis. Interestingly, a previous study had reported that the relative levels of FBP in liver doubled after only 1 h of anoxia in *T. s. elegans* [25]. When considering the presence of large liver glycogen stores and general increase in anaerobic glycolysis seen during anoxic exposure, the increase in FBP levels suggests the regulatory importance of aldolase in anoxic liver tissue. In addition, turtle liver PFK, FBPase and aldolase activities are nearly equivalent (0.68±0.044, 0.77±0.060 and 0.84±0.067 units/gram wet mass, respectively). This finding is in surprising as it is common for aldolase activity to be significantly greatly than that of either PFK or FBPase (11). The similarity between these activities may suggest a highly coordinated regulatory system at the F6P/FBP locus to regulate glycolytic vs. gluconeogenic flux in the turtle liver. An increase in FBP affinity may be necessary to deal with the rapid increases in substrate levels. As FBP is used as a substrate by many biological processes (glycolysis, gluconeogenesis, and the pentose phosphate pathway), an increased affinity for FBP by liver aldolase under anoxia could create the necessary driving force to ensure that more FBP would be directed toward glycolysis and away from other cellular processes during anoxia in the turtle.

The typical aldolase enzyme from liver tissue (ALDOB) has been kinetically characterized in many different organisms. Interestingly, the K_m_ values for F1P of turtle aldolase were not different between normoxic and anoxic states (both ∼14 mM) and were similar to the published value for humans (16 mM) [26]. In previous studies, the K_m_ for FBP of liver aldolase has been determined to range between 900 nM in rabbits and squirrels [27], [28] and 1600 nM in humans and fish [29], [30]. In this study, the K_m_ of turtle liver aldolase for FBP was found be approximately one order of magnitude lower than previously established values. Furthermore, the K_m_ for FBP significantly decreased from 240±30 nM in normoxic controls to 180±20 nM in anoxic liver, a 25% reduction, which is in line with previously recorded changes in kinetic parameters associated with enzymatic regulation of turtle liver glycolysis [9]. The lowest reported K_m_ of FBP for aldolase was 200 nM and was the result of a unique heterotetramer hybrid of corn (ALDOB) and rabbit (ALDOA) [31]. Interestingly, the kinetics of turtle liver aldolase were similar to those of the previously characterized corn:rabbit hybrid. This hybrid form of aldolase is of particular interest since both ALDOA and ALDOB were identified by mass spectrometry, immunoblotting and co-immunoprecipitation in turtle liver, suggesting the possibility that turtle liver aldolase exists as a natural heterotetramer.

The typical mammalian expression of ALDOA is restricted to skeletal muscle and kidney tissue, with expression in liver occurring only during early development [13]. However, the presence of both ALDOA and ALDOB in the liver tissue of turtles, as confirmed by this study, has been previously documented (parental α_4_ α_3_β_1_ α_2_β_2_ α_1_β_3_ parental β_4_) [15]. The aldolase α-subunits have a 50× higher enzymatic activity than the β-subunits, making hybrid forms of aldolase (α_3_β_1_ α_2_β_2_ α_1_β_3_) more active than the homotetramers of ALDOB found in the liver of mammals [16]. Given that the turtle experiences periodic exposure to low oxygen levels, including during prolonged dives and winter hibernation, the presence of a more active aldolase makes sense. If turtles enter into an oxygen deprived state, increases in anaerobic glycolysis may be facilitated, in-part, by a lower K_m_ of aldolase. Previous studies with anoxic red-eared slider turtles have identified an increase in aldolase activity after 5 h anoxia in heart, skeletal muscle, brain and kidney [32]. This present study demonstrates the presence of a different strategy in liver, an aldolase heterotetramer with higher enzymatic activity, although analysis of the co-immunoprecipitation results suggested that the relative α/β ratio does not significantly change with the onset of anoxia. This may suggest alternate mechanisms of aldolase regulation acting to regulate the enzyme’s kinetics during anoxia. To explore this possibility, we compared the structure of turtle aldolase to that of the well-characterized rabbit aldolase structures. Structural adaptations may predispose turtle aldolase with a greater ability to metabolize substrate during periods of oxygen restriction.

To explore the possibility that turtle liver aldolase may have structural features that allow greater rates of substrate conversion, the protein structures of both ALDOA and ALDOB were predicted from primary amino acid sequences using the SWISS-MODEL server. Protein models of both ALDOA and ALDOB were highly similar between the turtle enzymes and the crystal structures of rabbit aldolases. Next, docking analysis was used to determine if any alterations in the active site could provide a mechanism to explain the significantly higher affinity for FBP of the turtle enzyme. Interestingly, various differences between the interactions of FBP and aldolases (both ALDOA and ALDOB) in the turtle were present when compared to the crystal structures of the respective rabbit aldolase (Figure S2). It is unclear at this time whether these differences are able to confer physiological significance, but they do provide an additional mechanism for the lower K_m_ for FBP seen for turtle liver aldolase.

Results from this study suggest that turtle liver aldolase has an overall greater affinity for FBP than previously published models, suggesting that turtle liver aldolase facilitates the cleavage of FBP to DHAP and G3P more easily than many other vertebrate models. An increase in FBP levels during anoxia was found to be accompanied by both an increase in endogenous ALDOA and ALDOB protein expression, along with higher affinity for FBP by aldolase in turtle liver. This may ensure that FBP is directed towards glycolysis and away from other less essential cellular processes during anoxia. Additionally, evidence suggests that unique mechanisms may regulate liver aldolase in *T. s. elegans*. These mechanisms of aldolase regulation make sense given the need for increased reliance on glycolysis to help support ATP levels under anoxic conditions.

## Supporting Information

Figure S1 **Elution profiles for the purification of liver aldolase activity from normoxic** ***T. s. elegans*** **.** Purification of aldolase was carried out on A) DEAE^+^, B) Cibacron blue eluted with 5 mM FBP, and C) Cibacron blue eluted with a gradient of 0–2 M KCl.
(TIFF)

Figure S2 **Schematic representation of the predicted interactions between the ligand FBP and the active sites of aldolase.** Ligand interactions are modeled for the active site of A) turtle ALDOA compared to the crystal structures of B) rabbit ALDOA. Interactions between FBP and the active site of ALDOB were also modeled for C) turtle and the crystal structure for D) rabbit ALDOB. Interactions were modeled using MOE (v.2011.10).
(TIFF)
