# Supplementary figures and images for: Characterization of Fructose-1,6-Bisphosphate Aldolase during Anoxia in the Tolerant Turtle, Trachemys scripta elegans: An Assessment of Enzyme Activity, Expression and Structure
Source: PLoS One. 2013 Jul 18;8(7):e68830. doi: 10.1371/journal.pone.0068830 (PMC3715522; doi:10.1371/journal.pone.0068830)

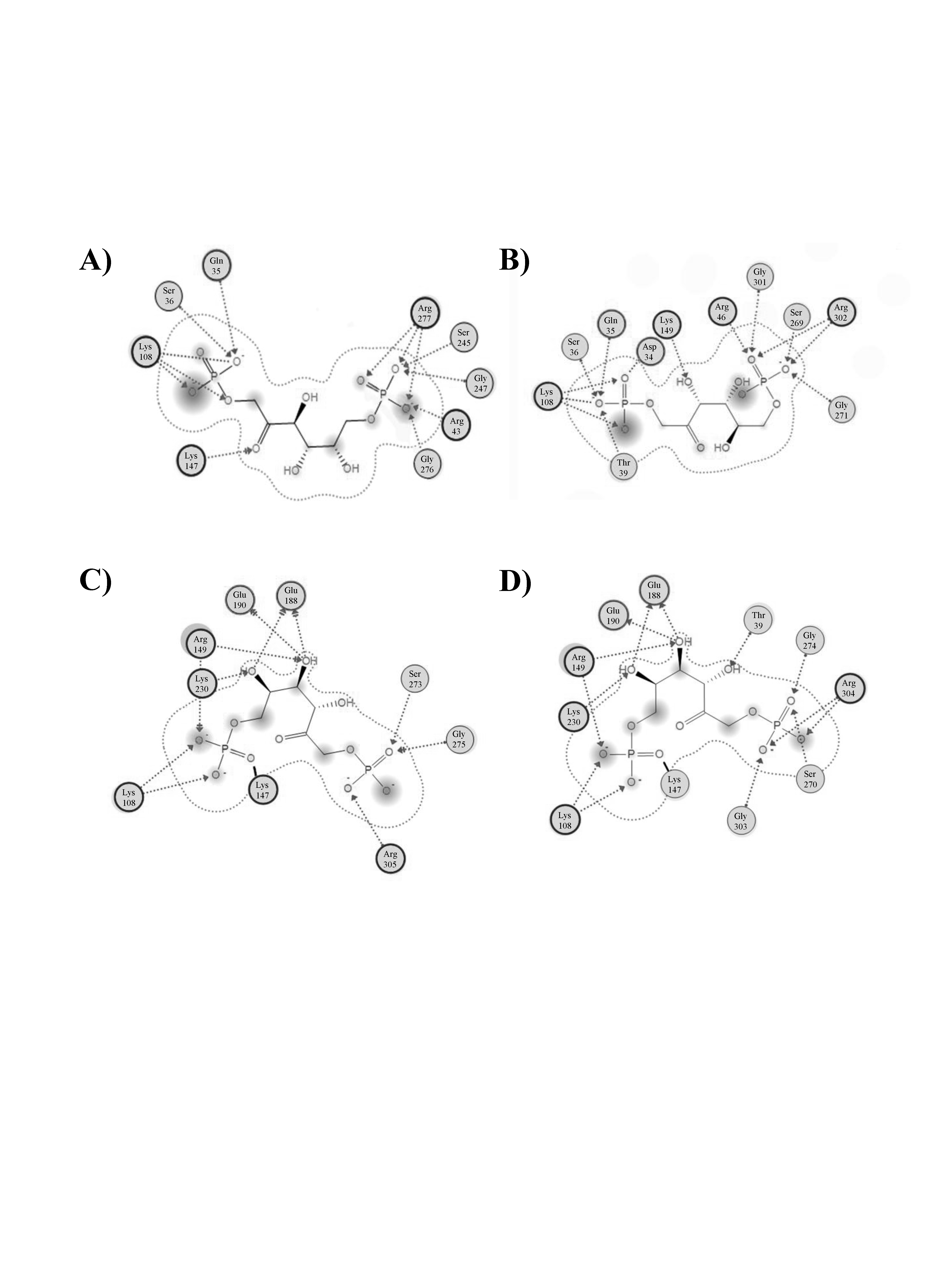

Supplement: Figure S2 — Schematic representation of the predicted interactions between the ligand FBP and the active sites of aldolase. Ligand interactions are modeled for the active site of A) turtle ALDOA compared to the crystal structures of B) rabbit ALDOA. Interactions between FBP and the active site of ALDOB were also modeled for C) turtle and the crystal structure for D) rabbit ALDOB. Interactions were modeled using MOE (v.2011.10). (TIFF) [file pone.0068830.s002.tiff]
